# Characterization and redox mechanism of asthma in the elderly

**DOI:** 10.18632/oncotarget.7075

**Published:** 2016-01-29

**Authors:** Li Zuo, Benjamin K. Pannell, Zewen Liu

**Affiliations:** ^1^ Radiologic Sciences and Respiratory Therapy Division, School of Health and Rehabilitation Sciences, The Ohio State University College of Medicine, Columbus, OH, USA; ^2^ The Interdisciplinary Biophysics Graduate Program, The Ohio State University, Columbus, OH, USA; ^3^ Department of Anesthesiology, Affiliated Ezhou Central Hospital, Renmin Hospital of Wuhan University Medical School, Hubei, China

**Keywords:** aging, allergic, inflammation, oxidative stress, respiratory

## Abstract

Asthma is a chronic disease characterized by reversible airflow limitation, coughing, bronchial constriction, and an inflammatory immune response. While asthma has frequently been categorized as emerging in childhood, evidence has begun to reveal that the elderly population is certainly susceptible to late-onset, or even long-standing asthma. Non-atopic asthma, most commonly found in elderly patients is associated with elevated levels of serum and sputum neutrophils and may be more detrimental than atopic asthma. The mortality of asthma is high in the elderly since these patients often possess more severe symptoms than younger populations. The redox mechanisms that mediate inflammatory reactions during asthma have not been thoroughly interpreted in the context of aging. Thus, we review the asthmatic symptoms related to reactive oxygen species (ROS) and reactive nitrogen species (RNS) in seniors. Moreover, immune status in the elderly is weakened in part by immunosenescence, which is broadly defined as the decline in functionality of the immune system that corresponds with increasing age. The effects of immunosenescence on the expression of biomarkers potentially utilized in the clinical diagnosis of asthma remain unclear. It has also been shown that existing asthma treatments are less effective in the elderly. Thus, it is necessary that clinicians approach the diagnosis and treatment of asthmatic senior patients using innovative methods. Asthma in the elderly demands more intentional diagnostic and therapeutic research since it is potentially one of the few causes of mortality and morbidity in the elderly that is largely reversible.

## INTRODUCTION

Asthma is a chronic lung disease of inflammation in the lower airways. Significant characteristics of asthma include bronchial hyper-reactivity, reversible airflow obstruction, and tissue remodeling [1]. Asthmatic patients frequently suffer from symptoms such as recurrent coughing, dyspnea, chest tightness, shortness of breath and sporadic wheezing [2, 3]. According to the World Health Organization, 4.3% of adults around the globe are diagnosed with asthma [4]. In the United States, the total number of people with asthma was estimated to be around 26 million in 2010 [4]. As to 2009, the age-adjusted prevalence of this disease increased markedly from 7.3 to 8.2% [5]. In addition, the death rates among patients with asthma are higher in adults compared to children [4]. The significance of this disease is already well known in the scientific and medical community. However, until recently the management of asthma has primarily been focused on younger populations. The elderly population continues to suffer from a higher mortality rate compared to children and younger adults due to the underdiagnosis and undertreatment of their asthma [6]. Many of the physiological alterations that occur in asthma and other lungs diseases have been observed in the aging lungs, suggesting that aging may be a determinant in the pathogenesis of lung disease [7, 8]. Specifically, fibrosis is mostly accompanied with aged organs and related pathologies such as wound healing in the lungs. For example, idiopathic pulmonary fibrosis (IPF), one of the most common pulmonary fibrosis diseases, progresses markedly with aging, and thus over two-thirds of IPF cases occur in seniors over 60 years old [8].

Despite this, very few studies have investigated the effects of aging on the pathogenesis of asthma [9]. This current review aims to highlight the significant impact of asthma on the elderly population, an area that has not been thoroughly reviewed. In addition, the role of reactive oxygen species (ROS)-induced oxidative stress in the pathogenesis of asthma is discussed. The unique challenges that medical professionals and researchers face when diagnosing and treating the elderly asthmatic population are examined.

## CHARACTERIZATION OF ASTHMA

As a prevalent inflammatory airway disease, asthma causes a significant public health burden and can manifest itself in any age. Different types of asthma have been distinguished. In order to be clinically effective in treating asthma, it is imperative to determine the specific type of asthma. For instance, allergic asthma, also known as atopic asthma, is commonly characterized by type 2 helper T cell (Th2) cytokine-induced eosinophilic inflammations in the airway [10, 11]. Studies have revealed that there is a strong link between genetic predisposition and early-onset of asthma [11]. For instance, Moffatt et al. revealed that the chromosome 17q locus has a significant effect on childhood-onset asthma [12]. Allergic asthma commonly starts at a young age and may either remit or recur in adulthood [13]. Allergic asthma is characterized by mast cell degranulation, amplified goblet cell hyperplasia, thickening of the sub-epithelial basement membrane, and epithelial damage [1]. On the contrary, non-atopic asthma, also known as non-allergic asthma, is associated with elevated levels of serum and sputum neutrophils and is most commonly found in elderly patients with a late-onset of the disease [14]. Interestingly, in terms of lung function, non-atopic asthma may be even more detrimental than atopic asthma [14]. A clinical study comparing atopic and non-atopic asthmatics found that patients with non-atopic asthma had lower forced expiratory volume in 1 second (FEV_1_) levels and more persistent symptoms than those with atopic asthma [15]. Meanwhile, age-related changes in the respiratory system can coincide with asthma and may contribute to the disease expression. Reduced diaphragmatic force generation and systemic inflammatory changes may arise in the elderly and intensify asthmatic phenotypes [6]. Elderly asthmatics have low serum immunoglobulin E (IgE) levels due to immunosenescence. Therefore, the measurement of total serum IgE for clinical asthma diagnosis, is less effective in elderly patients because the lower IgE levels can diminish the sensitivity of the test [14]. Unlike allergic asthma (characterized by a Th2-type inflammatory response), non-allergic asthma is more related to a Th1-type response, which causes neutrophils to increase [14].

The presence of airway inflammation in asthma typically relies on biomarkers found in bronchial biopsies and bronchial alveolar lavage [14, 16]. However, these techniques are complicated and invasive. Alternatively, the fraction of exhaled nitric oxide (FeNO) is a noninvasive method which can be used to indirectly determine the degree of inflammation in the airway [14]. However, it should be considered that some noninvasive measures, such as measurement of serum IgE levels, may be less sensitive when used to measure airway inflammation in elderly asthmatics [14].

## EPIDEMIOLOGY OF ASTHMA IN THE ELDERLY

**Figure 1:**
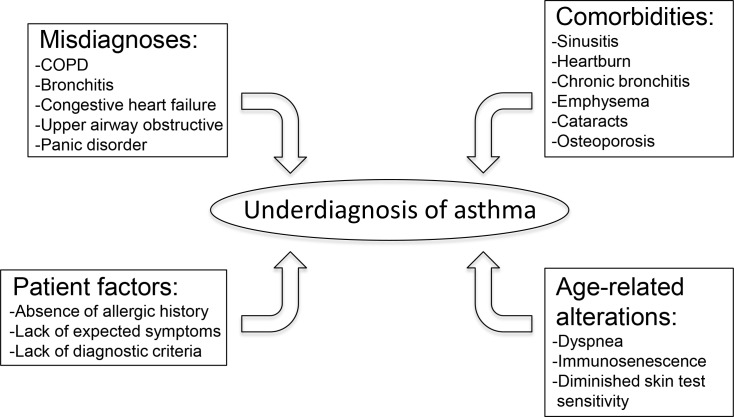
This schematic highlights the common factors that lead to underdiagnosis of asthma in the elderly population Abbreviations: COPD (chronic obstructive pulmonary disease).

Adult-onset asthma has become much more prevalent recently and is now a major public health concern in many countries due to its severity and lower remission rate [13]. The mortality of this disease is high in the elderly population [17]. Senior patients with asthma typically possess more severe symptoms than younger populations, which may require emergency treatment or hospital admission [3]. Unfortunately, asthma in the elderly can be misdiagnosed or underdiagnosed due to the under-reporting of symptoms, atypical presentation, or age-related factors (Figure 1) [2, 18]. For example, dyspnea, which is a common symptom in asthmatics, can be perceived as merely an age-related reduction in respiratory efficiency (Figure 1) [2]. Similarly, immunosenescence, which refers to age-related alterations in the immune system, can make asthma in the elderly challenging to diagnose since certain asthmatic phenotypes (e.g. increased serum neopterin) may increase with age in the absence of the disease (Figure 1) [14]. In addition, poor short-term responses to bronchodilators, absence of previous allergic diseases in patient history, diminished skin test sensitivity, lack of expected daytime/nighttime symptoms, and a lack of recognized criteria for diagnosing asthma in older adults all contribute to the underdiagnosis of asthma in the elderly (Figure 1) [19-21]. Furthermore, it is important to distinguish asthma from other airway diseases that share similar characteristics when making a diagnosis. For example, distinguishing COPD from asthma is even more difficult in older patients since both diseases are characterized by airway obstruction [2]. A large number of neutrophils are associated with both COPD and non-atopic asthma [14, 22, 23]. One study showed that among elderly asthmatic patients, 19.5% of cases received an improper diagnosis of COPD, while 27.3% of them received no diagnosis of asthma [19]. Moreover, it has been observed that asthmatic symptoms in the elderly have been falsely attributed to comorbid conditions such as congestive heart failure, COPD, or coronary artery disease (Figure 1) [6, 19]. While asthma and COPD are traditionally considered distinct diseases, clinicians are facing the challenge of treating patients that exhibit clinical features of both COPD and asthma. This is termed the asthma-COPD overlap syndrome (ACOS) [24]. ACOS is currently a much debated topic and little consensus has been established on a precise definition for ACOS. Thus, there are no established clinical guidelines to treat this patient population [25]. For instance, it is unknown if a treatment should be used that is outside of what is normally prescribed for COPD or asthma [26]. Experts disagree whether or not ACOS is the result of asthma and COPD existing simultaneously or if common pathogenic mechanisms are ultimately responsible [26]. The mixed phenotype of ACOS highlights an important area of research as a large percentage of adults with obstructive lung disease belong in this category [25]. In addition, the distinction between COPD and asthma in seniors is not easily recognized due to the common clinical presentations including airway inflammation or obstruction [13]. Thus, aging and its associated decline in respiratory function are being considered major factors in making it difficult to distinguish between asthma and COPD, thus contributing to this overlap syndrome [26]. To overcome these limitations, Gibson et al. proposed a multidimensional model focusing on four domain-assessments including self-management, risk factors, airways, and comorbidity [6]. Although this model has provided an effective approach, larger sample sizes are still needed in further studies [27].

Elderly asthmatics have more comorbid conditions and respiratory symptoms than young patients. These conditions include emphysema, heartburn, congestive heart failure, chronic bronchitis, sinusitis, and a history of smoking (Figure 1) [17]. In a study conducted by Piipari et al., they found that current smokers and ex-smokers had a significantly higher risk of developing asthma compared with those who have never smoked [28]. They concluded that smoking highly increases the risk of asthma in adulthood [28, 29]. Meanwhile, smoking can contribute to misdiagnosis and lead physicians to diagnose COPD rather than asthma, or to attribute symptoms to smoking rather than asthma [19]. Comorbidities, such as obesity and heart disease, can confound and complicate asthma and leave it underdiagnosed or hard to treat [6, 30]. Moreover, drugs targeting these comorbidities may interfere with asthma medications and exacerbate asthma in the elderly [30, 31].

## REDOX MECHANISMS IN THE PATHOGENESIS OF ASTHMA

It is generally agreed that increased reactive oxygen species (ROS) levels strongly correlate with the severity of asthma in patients [32, 33]. These higher amounts of ROS are largely responsible for the airway inflammation observed in asthma [34]. Previous literature has demonstrated the significant role that ROS and reactive nitrogen species (RNS) play during airway inflammation [35]. ROS/RNS initiate the inflammatory response in the lungs by activating nuclear factor-kappa B (NF-κB), mitogen activated protein kinase (MAPK), activator protein-1 (AP-1), and other transcription factors [35-37]. These redox-sensitive transcription factors promote the expression of numerous pro-inflammatory cytokines such as tumor necrosis factor (TNF)-α, interleukin (IL)-1, IL-6, and IL-8 [38], which subsequently induce the activation of inflammatory cells in the respiratory tract [35]. Interestingly, these inflammatory cells including macrophages, eosinophils, neutrophils, and monocytes have been shown to generate ROS themselves in order to kill the invading bacteria [32]. Age-related increases in ROS have resulted in oxidative damage to intracellular components [39, 40]. There is evidence demonstrating a strong correlation between *in vivo* oxidative damage and biological aging [39]. For example, investigators have observed that aging is associated with elevated ROS levels and a corresponding increase in oxidative stress throughout the body [39]. Aged organisms show an increase in oxidative damage to proteins [41], DNA [42, 43], and lipids [44, 45]. In addition, Zhang et al. revealed that certain redox-sensitive transcription factors (e.g. AP-1 and NF-κB) may have altered DNA binding activities that are associated with aging [39, 46]. Thus, we speculate that asthmatic symptoms related to oxidative stress in seniors may be significantly affected by age-associated ROS.

Additionally, oxidative stress and the resulting cellular modifications are prevalent in asthmatic patients [32, 33]. For instance, a study by Cassino et al. suggested that distal airway and alveolar tissue inflammation, such as that caused by eosinophils and macrophages, contributes to the structural changes (e.g. loss of elastic recoil) in elderly asthmatic patients [47]. Elevated amounts of myeloperoxidase (MPO) and eosinophil peroxidase (EPO) correspond with the increased numbers of neutrophils and eosinophils, respectively [33]. These biomarkers can be found in higher than normal amounts in the blood, sputum, and bronchoalveolar lavage fluid of asthmatic patients [33]. Increased lipid peroxidation is another harmful effect of oxidative stress that manifests itself in the lungs of asthma patients [16, 48]. Lipid peroxidation can generate ethane as a byproduct of a ROS chain reaction during asthma [49]. A study by Paredi et al. measured the level of ethane in the exhaled air of asthma patients [48]. It was demonstrated that lipid peroxidation was greater in patients with more severe asthma. This was confirmed by a tendency of patients with lower FEV_1_ to have increased exhaled ethane [48]. Since elderly asthmatic patients typically have decreased FEV_1_ due to severe obstructions in the airways [2], it is necessary that future studies elucidate the linkage between the concentration of exhaled ethane and asthma progression [48]. Undoubtedly, the deleterious effects of oxidative stress play a significant role in all cases of asthma. However, as oxidative stress has been shown to have increasing effects with age [50], it is reasonable to speculate that elderly patients with long-standing asthma are subjected to an even higher degree of oxidative cellular damage. Therefore, further research is needed to elucidate redox mechanisms that pertain to the progression of asthma in the elderly.

In recent history, RNS have been increasingly implicated in the damage of the airways [1]. Nitric oxide (NO) is synthesized by a group of NO synthase isoforms including inducible (iNOS), endothelial (eNOS), and neuronal (nNOS) [51]. The nNOS and eNOS are constitutively expressed in inhibitory nonadrenergic noncholinergic (iNANC) neurons in the airways. The iNANC neural pathway is responsible for bronchodilation [51]. Maarsingh et al. demonstrated in guinea pigs that a reduction in NO derived from nNOS and eNOS resulted in decreased relaxation of smooth muscle in the airway [51]. NO derived from iNOS is generated in epithelial cells and macrophages via cytokine induction during airway inflammation [51-53]. Therefore, given that airway inflammation is commonly observed in asthmatics, it is not surprising that increased NO levels are typically associated with the pathogenesis of asthma [53, 54]. NO is rather nonreactive and even participates in immune regulation [55]. However, in the presence of superoxide (O_2_^•-^), NO is converted to the much more reactive peroxynitrite (ONOO^−^) [54, 56]. As a powerful oxidant, ONOO^−^ has been shown to play a significant role in airway inflammation, airway epithelial damage, and airway hyperresponsiveness in a guinea pig model [57]. The presence of ONOO^−^ in the airways during inflammatory diseases may lead to the damage and loss of cells in the respiratory epithelium [58]. Furthermore, ONOO^−^ can induce tyrosine nitration, resulting in the stable end product 3-nitrotyrosine [54, 59]. 3-nitrotyrosine, as well as NO, can be quantified [60] and is a reliable indicator of nitrosative stress [1, 61]. A study by Lee et al. successfully utilized 3-nitrotyrosine, along with other oxidative products, as biomarkers of oxidative and nitrosative stress in a group of retired elderly coal miners [62].

**Figure 2:**
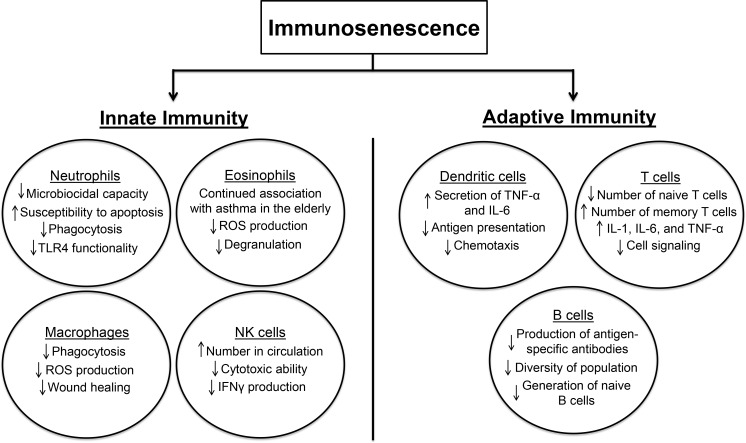
This schematic demonstrates the effect of immunosenescence on parts of the innate and adaptive immune systems Abbreviations: IFNγ (interferon-γ); IL (interleukin); NK (natural killer); ROS (reactive oxygen species); TLR (toll-like receptor); TNF-α (tumor necrosis factor-α).

Elderly adults are more predisposed to a variety of infections and diseases due, at least in part, to immunosenescence. The reduced ability of neutrophils to kill invading organisms is partially attributed to a decline in their ROS production [63]. Immunosenescence includes age-related functional declines in the innate and adaptive immune systems and is highlighted in Figure 2 [64-68]. However, the effects of immunosenescence on adaptive immunity are more well-known than in innate immunity [69]. Certain altered immune responses may facilitate the pathogenesis of asthma in the elderly [65]. Age-related altered functionality of immune response includes reduced levels and/or functionality of T cells, B cells, NK cells, NKT cells, eosinophils, and neutrophils (Figure 2) [66, 70, 71]. While naive T cells are typically seen to decrease with age, memory T cells are expected to increase presumably due to chronic viral stimulation from pathogens such as Epstein-Barr virus and cytomegalovirus (Figure 2) [65, 70]. Furthermore, the thymus, which is the main source of circulating T cells, decreases in size and activity with advanced age; accordingly, seniors are often less responsive to vaccinations or new infections compared to younger populations [72]. The predominant effect of aging on B cells is manifested in their poor ability to generate an antibody response [73]. The expression of toll-like receptors (TLRs) in monocytes and macrophages has been demonstrated to decrease with age (Figure 2) [65]. Consequently, downstream signaling that is mediated by TLRs is inhibited, and the risk of respiratory infection is increased [65]. An additional component of immunosenescence is increased systemic inflammation, sometimes referred to as “inflamm-aging.” Increased TNF-α and serum IL-6 are concomitant with inflamm-aging and may certainly be involved in the etiology of asthma in the elderly [63]. Age-related increases in the expression of TLR5 may also contribute to inflamm-aging in older people [74, 75].

As stated previously, endogenous ROS formation has clearly been demonstrated to increase during asthma. Thus, researchers have thoroughly investigated the link between oxidative stress and asthmatic symptoms, employing both human and animal studies [76]. For instance, a study conducted by Boldogh et al. revealed that inhibition of ROS produced by pollen NADPH oxidase prevented the immune inflammatory response in sensitized mice [77]. These results demonstrated that increased oxidative stress plays a pivotal role in the induction of the inflammatory immune response during asthma. Indeed, high levels of oxidative stress have been shown to result in an elevated amount of secreted signaling proteins such as pro-inflammatory cytokines [78]. Likewise, when the levels of oxidative stress are elevated to an extreme threshold, cell necrosis and apoptosis occur [78].

A logical question to ask is whether oxidative stress is a consequence of airway inflammation or is it an initiating factor in the development of the inflammation. The results of a study performed by Park et al. demonstrated that high amounts of oxidative stress occur before many of the common characteristics of allergic airway diseases including inflammation [79]. Epithelial shedding, smooth muscle contractions in the airway, and mucus hypersecretion all contribute to airway hyperresponsiveness induced by oxidative stress [80]. Accordingly, researchers have been able to mitigate the development of certain asthmatic symptoms via antioxidant treatments [79].

It has been speculated that certain individuals are genetically predisposed to severity of asthma due to the inability to resist oxidative stress [78, 81]. The reduced ability to scavenge ROS, due to a lowered antioxidant function, may facilitate airway inflammation and increase the risk of asthma [82, 83]. The glutathione S-transferase (GST) family of genes plays a pivotal role in the cellular protection against excessive ROS [82, 84]. Although Lee et al. determined that genotypic variants in the GST family increased the risk of asthma, they concluded that many gene-gene interactions were likely involved [82]. The need for further research on genetic determinants in the pathogenesis of asthma is crucial. However, many of the previous studies have focused on the development of asthma in children or younger adults [82, 85-87]. Thus, the need for similar genomic analysis on the elderly population is paramount.

Many of the physiological effects associated with oxidative stress that contribute to the pathogenesis of asthma are even more prominent in the elderly population. This is likely due in part to the progressive degenerative nature of this disease [88]. Chronic airway inflammation in elderly patients with long-standing asthma leads to a progressive loss of respiratory function [88]. Since ROS has previously been highlighted in this review as a major factor in airway inflammation, the implications of oxidative stress in elderly asthmatics are even more significant. Furthermore, we suggest that the impairment of FEV_1_ in the elderly with long-standing asthma could possibly be another indicator of the accumulation of oxidative damage (e.g. lipid peroxidation [48]) to the airways. It is well documented that there are certain structural and functional changes that occur in the respiratory system as age increases [89]. For instance, chest wall stiffness and reduced respiratory muscle strength in older people decrease the ability to exercise and increase the demand for oxygen at any level of exercise [89]. In addition, the number of alveoli is reduced with increasing age which has significant consequences on gas exchange. Therefore, it can be concluded that the natural aging process of the airways may exacerbate the deleterious effects experienced during asthma [89].

## DIAGNOSTIC CRITERIA AND THERAPEUTIC INTERVENTIONS IN THE ELDERLY WITH ASTHMA

Existing pharmacological therapies that are frequently utilized to treat and manage asthma include inhaled corticosteroids, β-agonists, and anti-IgE antibodies [2, 76]. Non-allergic asthma, which is more frequent in the elderly population, is less responsive to corticosteroids [14]. Total serum IgE measurement was initially thought to be a reliable indicator of asthma since many asthmatic patients are allergic and it might distinguish asthma from COPD during the diagnostic work-up [14]. However, it has been recognized that not all asthmatic patients are allergic. Furthermore, elderly patients tend to have lower IgE levels due to immunosenescence thus making the clinical diagnosis difficult using this method [14]. Much of the research that has investigated asthmatic biomarkers has excluded the elderly population. Thus, further studies are necessary in order to understand the effect of aging and immunosenescence on the expression of biomarkers that may be utilized in the clinical diagnosis of asthma [14].

Since the activity of oxidants in the airway during asthma play is prominent, non-invasive methods aimed at quantifying the stable end products of their reactive pathways have promising potential as indicators of airway oxidative stress [32, 33]. For instance, 3-nitrotyrosine and F_2_-isoprostanes have been detected in the exhaled air and urine, respectively, of asthma patients and are being evaluated for their efficacy in the diagnosis of asthma [90, 91]. Exhaled breath of asthmatic patients contains biomarkers, known as volatile organic compounds (VOCs), that can serve as indicators of asthma in children [92, 93]. Mass spectrometry and gas chromatography can individually detect these compounds in gas phase [94, 95]. Further research needs to be done to distinguish which biomarkers can be successfully utilized in asthma diagnosis in the elderly and how the VOC profile of elderly patients might be changed due to the aging process. Other methods for treating asthma, such as continuous positive airway pressure (CPAP) and bi-level positive airway pressure (BiPAP), have been proposed [96]. A study performed by Lafond et al. evaluated the efficacy of CPAP on patients (aged ≥ 18 yrs) with stable asthma and a diagnosis of obstructive sleep apnea (OSA). These two diseases may coexist, and certain problems reported in OSA (e.g. gastroesophageal reflux and nasal symptoms) may exacerbate asthma. The study determined that nocturnal CPAP did not improve certain parameters such as FEV_1_ or airway responsiveness; however, participants reported via questionnaires that their quality of life was measurably improved by CPAP at night [97]. Another study sought to determine if CPAP therapy could improve nocturnal asthmatic exacerbations in patients who had concomitant OSA. It was found that nocturnal symptoms improved in these patients without causing abnormalities in pulmonary function tests [98]. This suggests that OSA may contribute to nocturnal asthmatic exacerbations. Interestingly, there is evidence that CPAP may not have the same beneficial effect when administered to patients who do not concomitantly have OSA [96, 99]. Therefore, further large-scale clinical trials should be implemented to fully assess the value of CPAP/BiPAP as therapies for asthmatic patients with or without concomitant OSA. The implications of such studies may hold even greater significance for elderly asthmatic patients since CPAP/BiPAP are non-invasive treatments.

It has been demonstrated that current asthma treatments may be less effective in the elderly [34]. Older patients are less responsive to emergency bronchodilator therapy. Airway remodeling, concurrent medication, and comorbid conditions may all contribute to this effect [100]. Age-related increases in chest wall stiffness and residual volume due to decreased elastic recoil also play a role [101]. Another consideration is the change in pharmacokinetics as people get older. The timeline of drug absorption and metabolism is affected in the elderly [100]. Currently, inhaled corticosteroids continue to be one of the most commonly used treatments for the control of persistent asthmatic symptoms, even in the elderly [102, 103]. While the inhalation route offers a safe and effective treatment option for many asthma patients, ineffective inhalation technique is shown to increase with age. The frequency at which elders, at least 60 or 80 years of age, use inappropriate inhalation technique is as high as 40% and 60%, respectively [6]. Some of the identified factors leading to impaired inhalation in the elderly are impaired cognitive abilities and reduced inspiratory flow [6]. Currently, there is little information that sheds light on how reduced respiratory muscle strength in the elderly might affect aerosol distribution and particle deposition in the lung [89]. The long-term use of inhaled corticosteroids brings the risk of adverse effects such as skin thinning, bone loss, suppressed adrenal function, diabetes mellitus, pneumonia, and cataract formation [104, 105]. Many of these side effects are of particular importance to the elderly due to comorbidities and other age-related pathologies including increased cardiovascular risk and infections [106].

Inhaled β_2_-agonists and anticholinergics are also accepted treatments for the management of asthma [107, 108]. Long-acting β_2_-agonists (LABA) are used for moderate/severe asthma [109]. However, many clinical trials of LABAs have excluded subjects greater than 65 years old. Overall, very few clinical trials of asthma have focused on the elderly [109]. It has been shown that older people with cardiovascular comorbidities are at a greater risk for the negative cardiovascular effects of β_2_-adrenoceptor agonists [105, 109]. Therefore, it is urgent that trials continue to evaluate the safety and efficacy of accepted asthma treatments with specific focus on the elderly population.

Special care must be taken when prescribing asthma medications to the elderly. Adverse drug reactions are more common in the elderly population. This is due, in part, to factors such as co-administered drugs as well as altered pharmacodynamics and pharmacokinetics in the elderly [110]. Agents such as acetylsalicylic acid (ASA; aspirin) and nonsteroidal anti-inflammatory drugs (NSAIDs) are among the most frequently prescribed drugs in the elderly. It has been reported that patients simultaneously receiving NSAIDSs and corticosteroids might be at 15 times greater risk for peptic ulcer disease than those not receiving either drug [20]. Moreover, the use of self-prescribed ASA has risen and may go unrecognized. Therefore, it is important to closely examine medication history when treating elderly asthmatic patients [3]. Specific conditions often seen in the elderly, such as cataracts and osteoporosis (Figure 1), have also been linked to high doses of corticosteroids [20, 111, 112]. Estrogen replacement therapy has been used to help prevent osteoporosis in postmenopausal women [3]. However, estrogen use in postmenopausal women has been linked to an increased risk of developing asthma [113]. Vitamin D and calcium supplementation has been successfully utilized to help improve bone density in older patients that undergo long-term oral corticosteroid therapy [3, 114]. In addition, due to the role of oxidative stress in the pathogenesis of asthma, many studies have investigated the clinical benefits of antioxidant therapy. In general, the conclusions of these studies have been varied [115]. While low selenium levels have been associated with asthma, selenium supplementation has not been determined to be effective in treating asthma [116]. Randomized control trials investigated the supplementation of selenium [117] and vitamin E [118] in adults and found no clinical benefit for asthma. Alternatively, nuclear factor erythroid 2-related factor 2 (Nrf2) is a transcription factor involved in regulating important endogenous antioxidants and might be defective in severe asthmatics [119]. Certain Nrf2 activators, such as 1-(2-cyano-3-,12-dioxooleana-1,9-dien-28-oyl)imidazole-methyl ester, have been discovered and are being tested in clinical trials [119]. A study performed by Sussan et al. revealed that Nrf2 activation mitigates oxidative stress, alveolar destruction, and lung apoptosis induced by cigarette smoke exposure [120]. It is imperative that further research is performed to establish accepted guidelines for treatment of asthma in the elderly.

## CONCLUSION

The focus of this review is the potential influence of asthma in the elderly. Aging-induced inflammations could result in adaptive reduction of immune response known as immunosenescence [75]. The role of oxidative stress has previously been established in the pathogenesis of asthma. However, studies that investigate redox mechanisms in the asthmatic elderly are sparse. Historically, asthma in the elderly has been underdiagnosed due to many factors such as age-related declines in respiratory function as well as comorbid conditions. It is imperative that researchers and clinicians work together to establish guidelines for the diagnosis and treatment of asthma that address the specific needs of the elderly.

## References

[1] Zuo L, Koozechian MS, Chen LL (2014). Characterization of reactive nitrogen species in allergic asthma. Ann Allergy Asthma Immunol.

[2] Urso DL (2009). Asthma in the elderly. Curr Gerontol Geriatr Res.

[3] Boulet LP, Becker A, Berube D, Beveridge R, Ernst P (1999). Canadian Asthma Consensus Report, 1999. Canadian Asthma Consensus Group. CMAJ.

[4] Follenweider LM, Lambertino A (2013). Epidemiology of asthma in the United States. Nurs Clin North Am.

[5] Zahran HS, Bailey C, Garbe P (2011). Vital Signs: Asthma Prevalence, Disease Characteristics, and Self-Management Education-United States, 2001-2009 (Reprinted from MMWR, vol 60, pg 547-552, 2011). Jama-J Am Med Assoc.

[6] Gibson PG, McDonald VM, Marks GB (2010). Asthma in older adults. Lancet.

[7] King MJ, Hanania NA (2010). Asthma in the elderly: current knowledge and future directions. Curr Opin Pulm Med.

[8] Yanai H, Shteinberg A, Porat Z, Budovsky A, Braiman A, Zeische R, Fraifeld VE (2015). Cellular senescence-like features of lung fibroblasts derived from idiopathic pulmonary fibrosis patients. Aging (Albany NY).

[9] Inoue H, Niimi A, Takeda T, Matsumoto H, Ito I, Matsuoka H, Jinnai M, Otsuka K, Oguma T, Nakaji H, Tajiri T, Iwata T, Nagasaki T, Kanemitsu Y, Chin K, Mishima M (2014). Pathophysiological characteristics of asthma in the elderly: a comprehensive study. Ann Allergy Asthma Immunol.

[10] Bartemes KR, Iijima K, Kobayashi T, Kephart GM, McKenzie AN, Kita H (2012). IL-33-responsive lineage- CD25+ CD44(hi) lymphoid cells mediate innate type 2 immunity and allergic inflammation in the lungs. J Immunol.

[11] Wenzel SE (2012). Asthma phenotypes: the evolution from clinical to molecular approaches. Nat Med.

[12] Moffatt MF, Gut IG, Demenais F, Strachan DP, Bouzigon E, Heath S, von Mutius E, Farrall M, Lathrop M, Cookson WO, Consortium G (2010). A large-scale, consortium-based genomewide association study of asthma. N Engl J Med.

[13] Abramson MJ, Perret JL, Dharmage SC, McDonald VM, McDonald CF (2014). Distinguishing adult-onset asthma from COPD: a review and a new approach. Int J Chron Obstruct Pulm Dis.

[14] Rufo J, Taborda-Barata L, Lourenco O (2013). Serum biomarkers in elderly asthma. J Asthma.

[15] Knudsen TB, Thomsen SF, Nolte H, Backer V (2009). A population-based clinical study of allergic and non-allergic asthma. J Asthma.

[16] Fens N, Zwinderman AH, van der Schee MP, de Nijs SB, Dijkers E, Roldaan AC, Cheung D, Bel EH, Sterk PJ (2009). Exhaled breath profiling enables discrimination of chronic obstructive pulmonary disease and asthma. Am J Respir Crit Care Med.

[17] Diette GB, Krishnan JA, Dominici F, Haponik E, Skinner EA, Steinwachs D, Wu AW (2002). Asthma in older patients: factors associated with hospitalization. Arch Intern Med.

[18] Tzortzaki EG, Proklou A, Siafakas NM (2011). Asthma in the Elderly: Can We Distinguish It from COPD?. J Allergy.

[19] Bellia V, Battaglia S, Catalano F, Scichilone N, Incalzi RA, Imperiale C, Rengo F (2003). Aging and disability affect misdiagnosis of COPD in elderly asthmatics: the SARA study. Chest.

[20] Cardona V, Guilarte M, Luengo O, Labrador-Horrillo M, Sala-Cunill A, Garriga T (2011). Allergic diseases in the elderly. Clin Transl Allergy.

[21] Scichilone N, Callari A, Augugliaro G, Marchese M, Togias A, Bellia V (2011). The impact of age on prevalence of positive skin prick tests and specific IgE tests. Respir Med.

[22] Athanazio R (2012). Airway disease: similarities and differences between asthma, COPD and bronchiectasis. Clinics (Sao Paulo).

[23] Buist AS (2003). Similarities and differences between asthma and chronic obstructive pulmonary disease: treatment and early outcomes. Eur Respir J Suppl.

[24] Bateman ED, Reddel HK, van Zyl-Smit RN, Agusti A (2015). The asthma-COPD overlap syndrome: towards a revised taxonomy of chronic airways diseases?. Lancet Respir Med.

[25] Bujarski S, Parulekar AD, Sharafkhaneh A, Hanania NA (2015). The asthma COPD overlap syndrome (ACOS). Curr Allergy Asthma Rep.

[26] Zeki AA, Schivo M, Chan A, Albertson TE, Louie S (2011). The Asthma-COPD Overlap Syndrome: A Common Clinical Problem in the Elderly. J Allergy.

[27] McDonald VM, Higgins I, Wood LG, Gibson PG (2013). Multidimensional assessment and tailored interventions for COPD: respiratory utopia or common sense?. Thorax.

[28] Piipari R, Jaakkola JJ, Jaakkola N, Jaakkola MS (2004). Smoking and asthma in adults. Eur Respir J.

[29] Stapleton M, Howard-Thompson A, George C, Hoover RM, Self TH (2011). Smoking and asthma. JABFM.

[30] Boulet LP, Boulay ME (2011). Asthma-related comorbidities. Expert Rev Respir Med.

[31] Yanez A, Cho SH, Soriano JB, Rosenwasser LJ, Rodrigo GJ, Rabe KF, Peters S, Niimi A, Ledford DK, Katial R, Fabbri LM, Celedon JC, Canonica GW, Busse P, Boulet LP, Baena-Cagnani CE (2014). Asthma in the elderly: what we know and what we have yet to know. World Allergy Organ J.

[32] Sahiner UM, Birben E, Erzurum S, Sackesen C, Kalayci O (2011). Oxidative stress in asthma. World Allergy Organ J.

[33] Comhair SA, Erzurum SC (2010). Redox control of asthma: molecular mechanisms and therapeutic opportunities. Antioxid Redox Signal.

[34] Zuo L, Otenbaker NP, Rose BA, Salisbury KS (2013). Molecular mechanisms of reactive oxygen species-related pulmonary inflammation and asthma. Mol Immunol.

[35] Rahman I, Biswas SK, Kode A (2006). Oxidant and antioxidant balance in the airways and airway diseases. Eur J Pharmacol.

[36] Rahman I, MacNee W (1998). Role of transcription factors in inflammatory lung diseases. Thorax.

[37] Li N, Nel AE (2006). Role of the Nrf2-mediated signaling pathway as a negative regulator of inflammation: implications for the impact of particulate pollutants on asthma. Antioxid Redox Signal.

[38] Henricks PA, Nijkamp FP (2001). Reactive oxygen species as mediators in asthma. Pulm Pharmacol Ther.

[39] Kregel KC, Zhang HJ (2007). An integrated view of oxidative stress in aging: basic mechanisms, functional effects, and pathological considerations. Am J Physiol Regul Integr Comp Physiol.

[40] Butler D, Bahr BA (2006). Oxidative stress and lysosomes: CNS-related consequences and implications for lysosomal enhancement strategies and induction of autophagy. Antioxid Redox Signal.

[41] Grune T, Merker K, Jung T, Sitte N, Davies KJ (2005). Protein oxidation and degradation during postmitotic senescence. Free Radic Biol Med.

[42] Hamilton ML, Van Remmen H, Drake JA, Yang H, Guo ZM, Kewitt K, Walter CA, Richardson A (2001). Does oxidative damage to DNA increase with age?. Proc Natl Acad Sci U S A.

[43] Short KR, Bigelow ML, Kahl J, Singh R, Coenen-Schimke J, Raghavakaimal S, Nair KS (2005). Decline in skeletal muscle mitochondrial function with aging in humans. Proc Natl Acad Sci U S A.

[44] Judge S, Jang YM, Smith A, Hagen T, Leeuwenburgh C (2005). Age-associated increases in oxidative stress and antioxidant enzyme activities in cardiac interfibrillar mitochondria: implications for the mitochondrial theory of aging. FASEB J.

[45] Wozniak A, Drewa G, Wozniak B, Schachtschabel DO (2004). Activity of antioxidant enzymes and concentration of lipid peroxidation products in selected tissues of mice of different ages, both healthy and melanoma-bearing. Z Gerontol Geriatr.

[46] Zhang HJ, Doctrow SR, Xu L, Oberley LW, Beecher B, Morrison J, Oberley TD, Kregel KC (2004). Redox modulation of the liver with chronic antioxidant enzyme mimetic treatment prevents age-related oxidative damage associated with environmental stress. FASEB J.

[47] Cassino C, Berger KI, Goldring RM, Norman RG, Kammerman S, Ciotoli C, Reibman J (2000). Duration of asthma and physiologic outcomes in elderly nonsmokers. Am J Respir Crit Care Med.

[48] Paredi P, Kharitonov SA, Barnes PJ (2000). Elevation of exhaled ethane concentration in asthma. Am J Respir Crit Care Med.

[49] Zhou M, Liu Y, Duan Y (2012). Breath biomarkers in diagnosis of pulmonary diseases. Clin Chim Acta.

[50] Merksamer PI, Liu Y, He W, Hirschey MD, Chen D, Verdin E (2013). The sirtuins, oxidative stress and aging: an emerging link. Aging (Albany NY).

[51] Maarsingh H, Leusink J, Bos IS, Zaagsma J, Meurs H (2006). Arginase strongly impairs neuronal nitric oxide-mediated airway smooth muscle relaxation in allergic asthma. Respir Res.

[52] Iovine NM, Pursnani S, Voldman A, Wasserman G, Blaser MJ, Weinrauch Y (2008). Reactive nitrogen species contribute to innate host defense against Campylobacter jejuni. Infect Immun.

[53] Wedes SH, Khatri SB, Zhang R, Wu W, Comhair SA, Wenzel S, Teague WG, Israel E, Erzurum SC, Hazen SL (2009). Noninvasive markers of airway inflammation in asthma. Clin Transl Sci.

[54] MacPherson JC, Comhair SA, Erzurum SC, Klein DF, Lipscomb MF, Kavuru MS, Samoszuk MK, Hazen SL (2001). Eosinophils are a major source of nitric oxide-derived oxidants in severe asthma: characterization of pathways available to eosinophils for generating reactive nitrogen species. J Immunol.

[55] Wink DA, Hines HB, Cheng RY, Switzer CH, Flores-Santana W, Vitek MP, Ridnour LA, Colton CA (2011). Nitric oxide and redox mechanisms in the immune response. J Leukoc Biol.

[56] Droge W (2002). Free radicals in the physiological control of cell function. Physiol Rev.

[57] Sadeghi-Hashjin G, Folkerts G, Henricks PA, Verheyen AK, van der Linde HJ, van Ark I, Coene A, Nijkamp FP (1996). Peroxynitrite induces airway hyperresponsiveness in guinea pigs in vitro and in vivo. Am J Respir Crit Care Med.

[58] Nabeyrat E, Jones GE, Fenwick PS, Barnes PJ, Donnelly LE (2003). Mitogen-activated protein kinases mediate peroxynitrite-induced cell death in human bronchial epithelial cells. Am J Physiol Lung Cell Mol Physiol.

[59] Daiber A, Daub S, Bachschmid M, Schildknecht S, Oelze M, Steven S, Schmidt P, Megner A, Wada M, Tanabe T, Munzel T, Bottari S, Ullrich V (2013). Protein tyrosine nitration and thiol oxidation by peroxynitrite-strategies to prevent these oxidative modifications. Int J Mol Sci.

[60] Ghosh S, Erzurum SC (2011). Nitric oxide metabolism in asthma pathophysiology. Biochim Biophys Acta.

[61] Kharitonov SA, Barnes PJ (2003). Nitric oxide, nitrotyrosine, and nitric oxide modulators in asthma and chronic obstructive pulmonary disease. Curr Allergy Asthma Rep.

[62] Lee JS, Shin JH, Hwang JH, Baek JE, Choi BS (2014). Malondialdehyde and 3-nitrotyrosine in exhaled breath condensate in retired elderly coal miners with chronic obstructive pulmonary disease. Saf Health Work.

[63] Mathur SK (2010). Allergy and asthma in the elderly. Seminars in respiratory and critical care medicine.

[64] Jing Y, Gravenstein S, Chaganty NR, Chen N, Lyerly KH, Joyce S, Deng Y (2007). Aging is associated with a rapid decline in frequency, alterations in subset composition, and enhanced Th2 response in CD1d-restricted NKT cells from human peripheral blood. Exp Gerontol.

[65] Busse PJ, Mathur SK (2010). Age-related changes in immune function: effect on airway inflammation. J Allergy Clin Immunol.

[66] Gomez CR, Nomellini V, Faunce DE, Kovacs EJ (2008). Innate immunity and aging. Exp Gerontol.

[67] Dunn-Walters DK, Ademokun AA (2010). B cell repertoire and ageing. Curr Opin Immunol.

[68] Pawelec G (2007). Immunosenescence comes of age. Symposium on Aging Research in Immunology: The Impact of Genomics. EMBO reports.

[69] Xu YP, Qi RQ, Chen W, Shi Y, Cui ZZ, Gao XH, Chen HD, Zhou L, Mi QS (2012). Aging affects epidermal Langerhans cell development and function and alters their miRNA gene expression profile. Aging (Albany NY).

[70] Sansoni P, Vescovini R, Fagnoni F, Biasini C, Zanni F, Zanlari L, Telera A, Lucchini G, Passeri G, Monti D, Franceschi C, Passeri M (2008). The immune system in extreme longevity. Exp Gerontol.

[71] Weksler ME, Szabo P (2000). The effect of age on the B-cell repertoire. J Clin Immunol.

[72] Appay V, Sauce D, Prelog M (2010). The role of the thymus in immunosenescence: lessons from the study of thymectomized individuals. Aging (Albany NY).

[73] Mehr R, Melamed D (2011). Reversing B cell aging. Aging (Albany NY).

[74] Qian F, Wang X, Zhang L, Chen S, Piecychna M, Allore H, Bockenstedt L, Malawista S, Bucala R, Shaw AC, Fikrig E, Montgomery RR (2012). Age-associated elevation in TLR5 leads to increased inflammatory responses in the elderly. Aging cell.

[75] Qian F, Guo X, Wang X, Yuan X, Chen S, Malawista SE, Bockenstedt LK, Allore HG, Montgomery RR (2014). Reduced bioenergetics and toll-like receptor 1 function in human polymorphonuclear leukocytes in aging. Aging (Albany NY).

[76] Jiang L, Diaz PT, Best TM, Stimpfl JN, He F, Zuo L (2014). Molecular characterization of redox mechanisms in allergic asthma. Ann Allergy Asthma Immunol.

[77] Boldogh I, Bacsi A, Choudhury BK, Dharajiya N, Alam R, Hazra TK, Mitra S, Goldblum RM, Sur S (2005). ROS generated by pollen NADPH oxidase provide a signal that augments antigen-induced allergic airway inflammation. J Clin Invest.

[78] Cho YS, Moon HB (2010). The role of oxidative stress in the pathogenesis of asthma. Allergy Asthma Immunol Res.

[79] Park CS, Kim TB, Lee KY, Moon KA, Bae YJ, Jang MK, Cho YS, Moon HB (2009). Increased oxidative stress in the airway and development of allergic inflammation in a mouse model of asthma. Ann Allergy Asthma Immunol.

[80] Hung CY, Huang FL, Shi LS, Ka SM, Wang JY, Tsai YC, Hung TJ, Ye YL (2013). The Ethanol Extract of Osmanthus fragrans Flowers Reduces Oxidative Stress and Allergic Airway Inflammation in an Animal Model. J Evid Based Complementary Altern Med.

[81] Yang IA, Fong KM, Zimmerman PV, Holgate ST, Holloway JW (2008). Genetic susceptibility to the respiratory effects of air pollution. Thorax.

[82] Lee YL, Hsiue TR, Lee YC, Lin YC, Guo YL (2005). The association between glutathione S-transferase P1, M1 polymorphisms and asthma in Taiwanese schoolchildren. Chest.

[83] Reddy PH (2011). Mitochondrial Dysfunction and Oxidative Stress in Asthma: Implications for Mitochondria-Targeted Antioxidant Therapeutics. Pharmaceuticals (Basel).

[84] Saadat M, Ansari-Lari M (2007). Genetic polymorphism of glutathione S-transferase T1, M1 and asthma, a meta-analysis of the literature. Pak J Biol Sci.

[85] Daniels SE, Bhattacharrya S, James A, Leaves NI, Young A, Hill MR, Faux JA, Ryan GF, le Souef PN, Lathrop GM, Musk AW, Cookson WO (1996). A genome-wide search for quantitative trait loci underlying asthma. Nature.

[86] Fryer AA, Bianco A, Hepple M, Jones PW, Strange RC, Spiteri MA (2000). Polymorphism at the glutathione S-transferase GSTP1 locus. A new marker for bronchial hyperresponsiveness and asthma. Am J Respir Crit Care Med.

[87] A genome-wide search for asthma susceptibility loci in ethnically diverse populations (1997). The Collaborative Study on the Genetics of Asthma (CSGA). Nat Genet.

[88] Weiner P, Magadle R, Waizman J, Weiner M, Rabner M, Zamir D (1998). Characteristics of asthma in the elderly. Eur Respir J.

[89] Berend N (2005). Normal ageing of the lung: implications for diagnosis and monitoring of asthma in older people. Med J Aust.

[90] Dworski R, Roberts LJ, Murray JJ, Morrow JD, Hartert TV, Sheller JR (2001). Assessment of oxidant stress in allergic asthma by measurement of the major urinary metabolite of F2-isoprostane, 15-F2t-IsoP (8-iso-PGF2alpha). Clin Exp Allergy.

[91] Paredi P, Kharitonov SA, Barnes PJ (2002). Analysis of expired air for oxidation products. Am Respir Crit Care Med.

[92] Dragonieri S, Schot R, Mertens BJ, Le Cessie S, Gauw SA, Spanevello A, Resta O, Willard NP, Vink TJ, Rabe KF, Bel EH, Sterk PJ (2007). An electronic nose in the discrimination of patients with asthma and controls. J Allergy Clin Immunol.

[93] Smolinska A, Klaassen EM, Dallinga JW, van de Kant KD, Jobsis Q, Moonen EJ, van Schayck OC, Dompeling E, van Schooten FJ (2014). Profiling of volatile organic compounds in exhaled breath as a strategy to find early predictive signatures of asthma in children. PloS one.

[94] Pauling L, Robinson AB, Teranishi R, Cary P (1971). Quantitative analysis of urine vapor and breath by gas-liquid partition chromatography. Proc Natl Acad Sci U S A.

[95] Moser B, Bodrogi F, Eibl G, Lechner M, Rieder J, Lirk P (2005). Mass spectrometric profile of exhaled breath--field study by PTR-MS. Respir Physiol Neurobiol.

[96] Yim S, Fredberg JJ, Malhotra A (2007). Continuous positive airway pressure for asthma: not a big stretch?. Eur Respir J.

[97] Lafond C, Series F, Lemiere C (2007). Impact of CPAP on asthmatic patients with obstructive sleep apnoea. Eur Respir J.

[98] Ciftci TU, Ciftci B, Guven SF, Kokturk O, Turktas H (2005). Effect of nasal continuous positive airway pressure in uncontrolled nocturnal asthmatic patients with obstructive sleep apnea syndrome. Respir Med.

[99] Martin RJ, Pak J (1991). Nasal CPAP in nonapneic nocturnal asthma. Chest.

[100] Banerji A, Clark S, Afilalo M, Blanda MP, Cydulka RK, Camargo CA (2006). Prospective multicenter study of acute asthma in younger versus older adults presenting to the emergency department. J Am Geriatr Soc.

[101] Reed CE (2010). Asthma in the elderly: diagnosis and management. J Allergy Clin Immunol.

[102] Schmier JK, Halpern MT, Jones ML (2005). Effects of inhaled corticosteroids on mortality and hospitalisation in elderly asthma and chronic obstructive pulmonary disease patients: appraising the evidence. Drug Aging.

[103] Scichilone N, Pedone C, Battaglia S, Sorino C, Bellia V (2014). Diagnosis and management of asthma in the elderly. Eur J Intern Med.

[104] Battaglia S, Cardillo I, Lavorini F, Spatafora M, Scichilone N (2014). Safety considerations of inhaled corticosteroids in the elderly. Drug Aging.

[105] Newnham DM (2001). Asthma medications and their potential adverse effects in the elderly: recommendations for prescribing. Drug safety.

[106] McDonough AK, Curtis JR, Saag KG (2008). The epidemiology of glucocorticoid-associated adverse events. Curr Opin Rheumatol.

[107] Lommatzsch M, Virchow JC (2014). Severe asthma: definition, diagnosis and treatment. Dtsch Arztebl Int.

[108] Beeh KM, Beier J (2009). Indacaterol, a novel inhaled, once-daily, long-acting beta2-agonist for the treatment of obstructive airways diseases. Adv Ther.

[109] Goeman DP, Douglass JA (2007). Optimal management of asthma in elderly patients: strategies to improve adherence to recommended interventions. Drug Aging.

[110] Ohnishi A, Kato M, Kojima J, Ushiama H, Yoneko M, Kawai H (2003). Differential pharmacokinetics of theophylline in elderly patients. Drug Aging.

[111] Weinstein RS (2011). Clinical practice. Glucocorticoid-induced bone disease. N Engl J Med.

[112] Wang JJ, Rochtchina E, Tan AG, Cumming RG, Leeder SR, Mitchell P (2009). Use of inhaled and oral corticosteroids and the long-term risk of cataract. Ophthalmology.

[113] Romieu I, Fabre A, Fournier A, Kauffmann F, Varraso R, Mesrine S, Leynaert B, Clavel-Chapelon F (2010). Postmenopausal hormone therapy and asthma onset in the E3N cohort. Thorax.

[114] Pitt P, Li F, Todd P, Webber D, Pack S, Moniz C (1998). A double blind placebo controlled study to determine the effects of intermittent cyclical etidronate on bone mineral density in patients on long-term oral corticosteroid treatment. Thorax.

[115] Kirkham P, Rahman I (2006). Oxidative stress in asthma and COPD: antioxidants as a therapeutic strategy. Pharmacol Ther.

[116] Allan K, Devereux G (2011). Diet and asthma: nutrition implications from prevention to treatment. J Am Diet Assoc.

[117] Shaheen SO, Newson RB, Rayman MP, Wong AP, Tumilty MK, Phillips JM, Potts JF, Kelly FJ, White PT, Burney PG (2007). Randomised, double blind, placebo-controlled trial of selenium supplementation in adult asthma. Thorax.

[118] Pearson PJ, Lewis SA, Britton J, Fogarty A (2004). Vitamin E supplements in asthma: a parallel group randomised placebo controlled trial. Thorax.

[119] Barnes PJ (2012). Severe asthma: advances in current management and future therapy. The J Allergy Clin Immunol.

[120] Sussan TE, Rangasamy T, Blake DJ, Malhotra D, El-Haddad H, Bedja D, Yates MS, Kombairaju P, Yamamoto M, Liby KT, Sporn MB, Gabrielson KL, Champion HC, Tuder RM, Kensler TW, Biswal S (2009). Targeting Nrf2 with the triterpenoid CDDO-imidazolide attenuates cigarette smoke-induced emphysema and cardiac dysfunction in mice. Proc Natl Acad Sci U S A.

